# Microneedle array facilitates hepatic sinusoid construction in a large-scale liver-acinus-chip microsystem

**DOI:** 10.1038/s41378-023-00544-w

**Published:** 2023-06-07

**Authors:** Shibo Li, Chengpan Li, Muhammad Imran Khan, Jing Liu, Zhengdi Shi, Dayong Gao, Bensheng Qiu, Weiping Ding

**Affiliations:** 1 Department of Electronic Engineering and Information Science, University of Science and Technology of China, Hefei, Anhui 230027 China; 2 Department of Oncology, the First Affiliated Hospital of USTC, Division of Life Sciences and Medicine, University of Science and Technology of China, Hefei, Anhui 230001 China; 3 Center for Biomedical Imaging, University of Science and Technology of China, Hefei, Anhui 230027 China; 4 School of Biology, Food and Environment, Hefei University, Hefei, Anhui 230601 China; 5 Department of Mechanical Engineering, University of Washington, Seattle, WA 98195 USA

**Keywords:** Microfluidics, Nanofabrication and nanopatterning

## Abstract

Hepatic sinusoids play a key role in maintaining high activities of liver cells in the hepatic acinus. However, the construction of hepatic sinusoids has always been a challenge for liver chips, especially for large-scale liver microsystems. Herein, we report an approach for the construction of hepatic sinusoids. In this approach, hepatic sinusoids are formed by demolding a self-developed microneedle array from a photocurable cell-loaded matrix in a large-scale liver-acinus-chip microsystem with a designed dual blood supply. Primary sinusoids formed by demolded microneedles and spontaneously self-organized secondary sinusoids can be clearly observed. Benefiting from significantly enhanced interstitial flows by formed hepatic sinusoids, cell viability is witnessed to be considerably high, liver microstructure formation occurs, and hepatocyte metabolism is enhanced. In addition, this study preliminarily demonstrates the effects of the resulting oxygen and glucose gradients on hepatocyte functions and the application of the chip in drug testing. This work paves the way for the biofabrication of fully functionalized large-scale liver bioreactors.

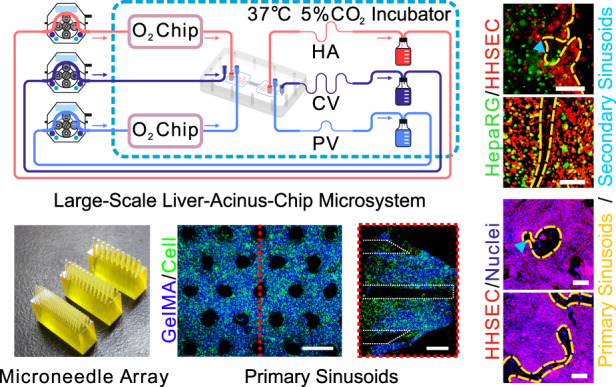

## Introduction

The liver is one of the most important organs in the human body and plays a key role in the metabolism of many substances, such as blood glucose and drugs^1,2^. To understand the complex liver structure and associated function at the microscale, researchers define two main divisions for liver microstructures, the hepatic lobule^3–5^ and hepatic acinus^6–8^. Compared with the hepatic lobule, a hexagonal division that relies mostly on the characteristics of liver microstructure, the hepatic acinus, a fusiform or olive-shaped structure centered on the portal area and approximately one-third of the volume of the hepatic lobule, is defined by both structural and functional properties^9^. In addition, the hepatic acinus is also more concise in structure^10^. In the hepatic acinus, blood enters the hepatic sinusoid via the portal vein (PV) and hepatic artery (HA) and exits via the central vein (CV)^6^. This unique dual blood supply with tri-vascular microcirculation spatially generates concentration gradients of both oxygen and nutrients (e.g., glucose) in the hepatic acinus^11^ and then significantly forms structure and function differences in various regions of the hepatic acinus, e.g., differences in metabolism^12^, liver gene expression^13^ and sinusoid morphology^14^. These differences may be magnified under varying physiological and pathological conditions^15^ or in the presence of drugs^16^.

To discover the mechanism of liver function, investigate the hepatotoxicity of drugs, and mimic liver-related diseases, animal models are routinely used^17^, but the application of animal models is limited due to interspecies differences in structure and function^18^. 2D and 3D cell cultures are alternatives to minimize the difference between human and nonhuman cells^19–21^. However, 2D culture is very different from in vivo 3D cell growth environments^22,23^, and traditionally proposed 3D culture only occasionally forms a few very simple structures^24^ and rarely generates self-organized perfusable vascular structures^25,26^, let alone a complex dual blood supply and tri-vascular system. Very recently, organ-on-a-chip techniques have undergone rapid development^27^ and enable the biomimicry of human tissue culture on a microfluidic platform^28–30^. In addition, the organ-on-a-chip technique can build microstructures and microenvironments more similar to physiological ones than traditionally developed 3D culture methods^24^.

As one of the main members of the organ-on-a-chip, although liver chips have always been focused extensively^31^, they are very different from in vivo livers, and they still present many challenges, especially in the construction of peripheral vascular systems^32^ and hepatic sinusoids^2,33,34^. In terms of the construction of hepatic sinusoids, initially reported chips achieved only 2D dynamic endotheliocyte culture; that is, endothelial cells are simply laid on the surface of the culture zone, and the culture medium flows through the cell surface^35–38^. Later, researchers directly constructed customized scaffold structures as sinusoids using approaches such as photolithography^39,40^, laser-based cavitation^41^, dielectrophoresis^42^, and magnetic field induction^43^. However, these approaches have limitations either in spatial resolution (usually >100 µm) or in structural complexity (it is difficult to form a complex 3D structure)^44^. Then, pioneering researchers tried to form sinusoids by the self-assembly of endotheliocytes with the help of growth-factor gradients^45–47^ or culture medium flows^48,49^. Physiologically similar sinusoids are the basis of large-scale liver tissue culture, as oxygen and nutrients can be transported to all cells^2^. However, the assembly process is slow, the likelihood of the formation of perfusable sinusoids is low, and the growth and migration of endotheliocytes are usually unordered, leading to poor reproducibility^41^.

In terms of the construction of the tri-vascular (HA–PV–CV) system for feeding cultured cells, many of the early liver chips neglected the tri-vascular structure and even the dynamic flow^50,51^, which has been proven to promote cell function and long-term culture^51,52^. Later, liver chips with a single-flow pathway as a vascular substitute emerged, supplying the cultured cells with oxygen and nutrients and removing waste^35,51,53–55^. However, it is difficult for the single-vascular structure to form physiologically similar oxygen and nutrient gradients in the cell culture area, which are considered one of the main factors resulting in the differentiated functional regions of hepatic acinus^56^. Furthermore, it is also difficult to reach large-scale tissues because of the transportation limitation of oxygen and nutrients. Currently, a dual-vascular structure, originally used for the organ-on-a-chip^57–59^, has been used for liver chips^60,61^. In this structure, the cell culture area is located between two fluid pathways, forming an artery-tissue-vein microcirculation system. This system can form microvascular networks and concentration gradients^39,60^. However, it still fails to achieve a double blood supply, as observed in the liver, much less physiologically similar oxygen and nutrient gradients^48,49^. There are two general design approaches to pursue the tri-vascular structure based on the two divisions for liver microstructures. Statistically, chips with the hepatic lobule design are in the majority, although the hepatic acinus design is simple, especially for the tri-vascular structure.

In our previous work, we proposed two designs of the dual blood supply tri-vascular structure for a single hepatic lobule chip and a multiple hepatic lobules chip^48,49^. For the single hepatic lobule chip, the structure is simple, and it only consists of 3 layers of polydimethylsiloxane (PDMS). This chip forms sinusoid-like structures^49^. However, the perfusable sinusoid is barely observed. The multiple hepatic lobule chip consists of 5 layers of polymethyl methacrylate (PMMA) and one layer of PDMS with continuously arranged hexagon areas. With the micropillar arrays specifically designed as PV, HA, and CV, this chip forms perfusable sinusoids via flow-guided self-assembly and replicates the convergence of venues from PV and arterioles from HA to the connection between sinusoids and CV^48^. However, the structure of the multiple hepatic lobule chip is very complicated, and the degree of formation of perfusable sinusoids is unsatisfactory. In summary, although the dual blood supply tri-vascular structure has already been developed, the formation of perfusable sinusoids for large-scale liver-tissue culture is still lacking^60–63^.

In this study, therefore, we present a microneedle-assisted hepatic sinusoid construction for a large-scale liver-acinus-chip microsystem with a dual blood supply. The research process is as follows: first, we designed a dual blood supply liver chip based on the hepatic acinus structure; then, we fabricated a microneedle array using 3D printing and promoted the formation of perfusable hepatic sinusoids by demolding a self-developed microneedle array from a photocurable cell-loaded matrix in the liver acinus chip; afterward, we investigated the effects of microneedle array-induced hepatic sinusoids on fluid flows inside the culture area, liver metabolism, cellular activity, and tissue formation; finally, we preliminarily demonstrated the effects of the resulting oxygen and glucose gradients on hepatocyte functions and the application of the chip in drug testing. This work provides insights into the construction of more biomimetic liver chips with sinusoids.

## Results

### Fabrication of the microneedle-assisted hepatic acinus chip (mHAC)

**Fig. 1:**
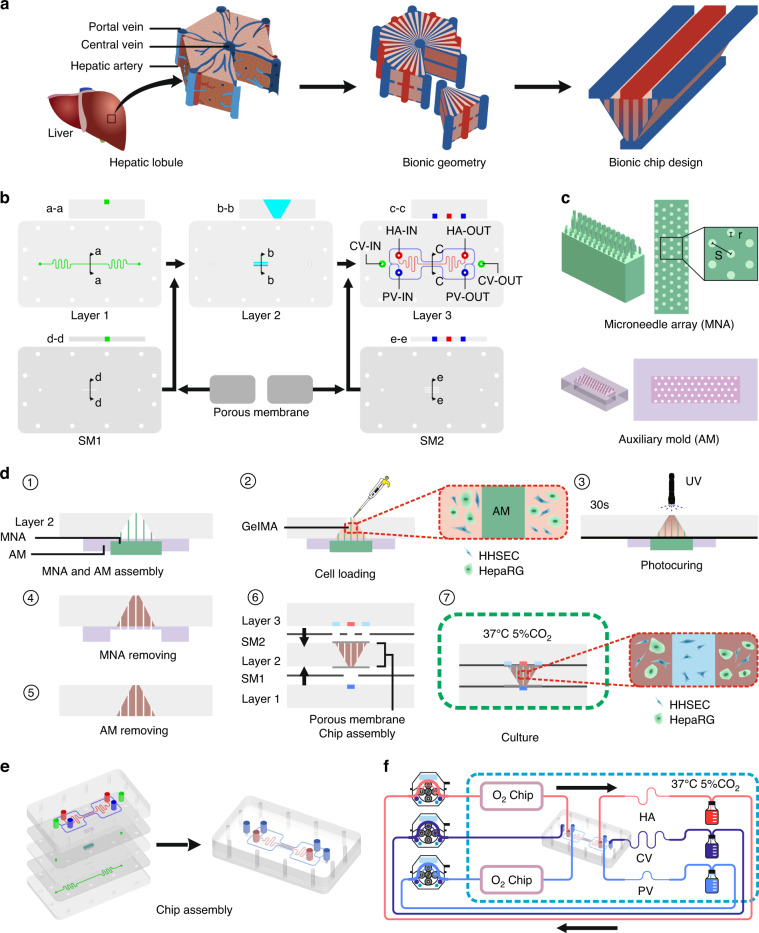
Design and operation of the mHAC. **a** Schematic of the hepatic acinus with the tri-vascular system and its design. **b** Schematic components of the mHAC. **c** Schematic of the microneedle array and its auxiliary mold for demolding. **d** Schematic of the assembly of the mHAC. **e** 3D schematic of the chip. **f** Experimental setup for the mHAC (the wavy line denotes the polytetrafluoroethylene (PTFE) tube, while the other line denotes the silicon tube)

The design of the cell culture area of the mHAC is inspired by the structure of the human hepatic acinus. That is, a single culture area is designed as a triangular prism, consisting of 1/6 of a hepatic lobule or 1/2 of a hepatic acinus (Fig. 1a). In the mHAC, the CV pathway is located on the lower edge of the triangular prism, while the PV pathway is located on the other two edges of the triangular prism, and the HA pathway is between the PV pathways. In the culture area, the hepatic sinusoids are considered to extend through the whole culture area, perpendicular to the top surface of the triangular prism (Fig. 1a). In the cultured chip, the culture media from PV and HA partially infiltrate into the culture area, and the culture medium flows through the cell-cultured area into the CV, as reported physiologically^11^.

The chip was designed as shown in Fig. 1b. It was composed of 3 layers of PMMA: Layer 1 was for CV (green), Layer 2 was for the culture area (light blue; isosceles trapezoid), and Layer 3 was for PV (blue) and HA (red). In addition, the layers were separated/sandwiched with a silicone membrane (SM) and a porous membrane (only for the culture area). The detailed design parameters (Fig. S1) and pictures of the chip (Fig. S2) are shown in the Supplementary Information. In addition, a microneedle array was designed to assist in the formation of the primary hepatic sinusoids (Fig. 1c and Fig. S3). Then, the chip was assembled according to the procedure in Fig. 1d. Finally, the assembled chip (Fig. 1e) was connected to a culture system (Fig. 1f and Fig. S4). In experiments, the perfusable chip microsystem could run stably for more than 14 days.

### Characterization of sinusoids formed by microneedles

**Fig. 2:**
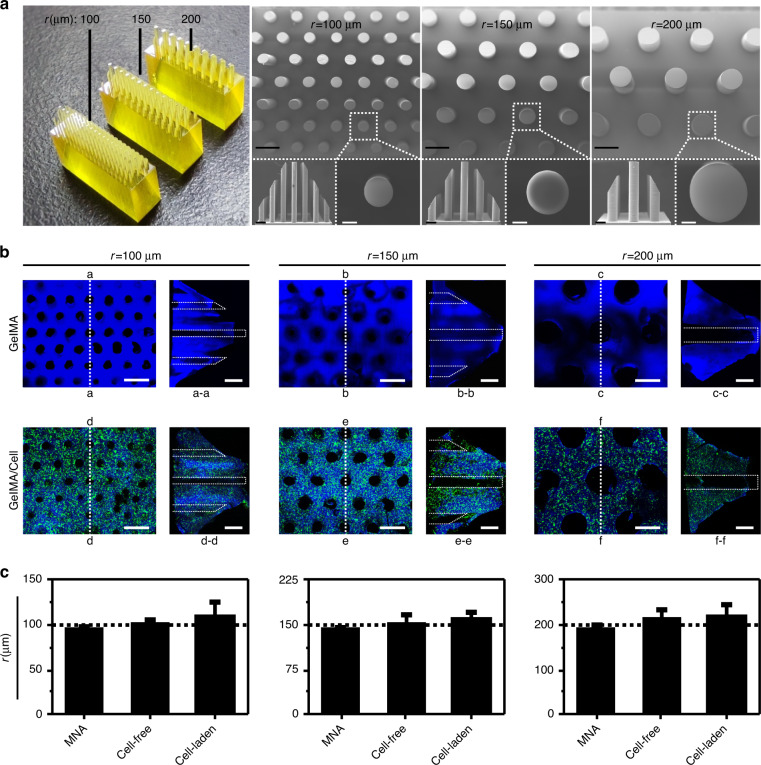
Characterization of sinusoids formed using microneedles. **a** 3D-printed microneedle array and scanning electron microscopy (SEM) images of the 3D-printed microneedle array. Black scale bar = 200 μm. White scale bar = 50 μm. **b** CLSM images of cell-free and cell-laden ECM with formed sinusoid pathways. Scale bar = 500 μm. **c** Sizes of the microneedle array, cell-free GelMA, and cell-laden GelMA

In this study, 3D printed microneedles with radii of 100, 150, and 200 μm were designed to inspire the generation of primary sinusoids (Fig. 2a; the volume fraction of microposts is designed to be close to the porosity of sinusoids). The confocal laser scanning microscopy (CLSM) results showed that after demolding, sinusoid pathways were formed well in both cell-free and cell-laden gelatin methacryloyl (GelMA) extracellular matrix (ECM) (Fig. 2b). Although small breakages may occur due to demolding damage, the majority of pathways are intact. In addition, the radius of the pathways is slightly larger than the radius of the microneedles, possibly resulting from the dehydration of GelMA during scanning with CLSM. Generally, the pathways for primary sinusoids were formed as expected (Fig. 2c).

### Microneedle-inspired liver chip increases flows inside the ECM

**Fig. 3:**
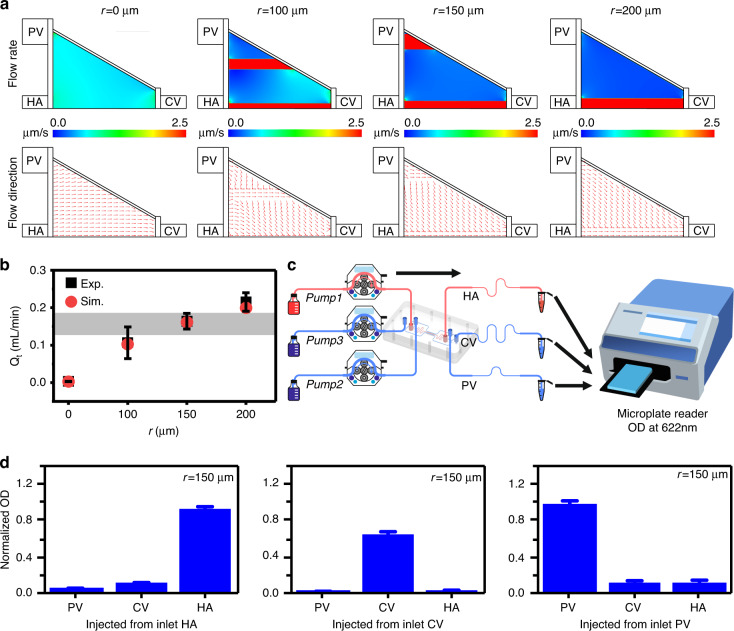
Flow analysis of HA, PV, and CV. **a** Flow simulation in the hepatic acinus. **b** Comparison of the flow rate across the culture areas (*Q*_*t*_) between theoretically simulated and experimentally measured results. **c** Schematic of the testing system for flow analysis. **d** Concentration of the dye in HA, PV, and CV, depicted as the normalized OD (622 nm) of the dye

To demonstrate the advantages of sinusoids formed using microneedles in terms of flows inside ECM, the effects of formed sinusoids on flows were theoretically and experimentally investigated. The simulation shows that the flow directions of fluids are mainly from PV and HA to CV via the formed sinusoids, with the convergence of parts of fluids from sinusoids to CV in the culture area (Fig. 3a). The flow rate across the culture area (*Q*_*CO*_ − *Q*_*CI*_ or *Q*_*HI*_ − *Q*_*HO*_ + *Q*_*PI*_ − *Q*_*PO*_) increases with the radius of the formed sinusoids, resulting from the decreasing flow resistance (Fig. 3b and Fig. S5). For formed sinusoids of radius 150 μm, the flow rate, which is close to the value experimentally reported in the literature, simulates the interstitial flow across hepatocytes more physiologically^64^. To experimentally confirm the flow directions of fluids in PV, HA, and CV, a blue dye was added in PV, HA, and CV separately (Fig. 3c). When the dye was injected from one of the vascular pathways (e.g., HA), the concentrations of the dye in the rest of the vascular pathways (e.g., PV and CV) were relatively negligible (Fig. 3d and Fig. S6). Thus, mixing of HA and PV beyond the culture area barely occurred, and the flow directions of fluids in the culture area were definitely from PV and HA to CV. In vivo, there are differences between HA and PV in terms of supplied oxygen and nutrients. These results also imply that differences in oxygen and nutrients can be maintained if applied.

### Microneedle-inspired liver chip heightens the metabolism of hepatocytes

**Fig. 4:**
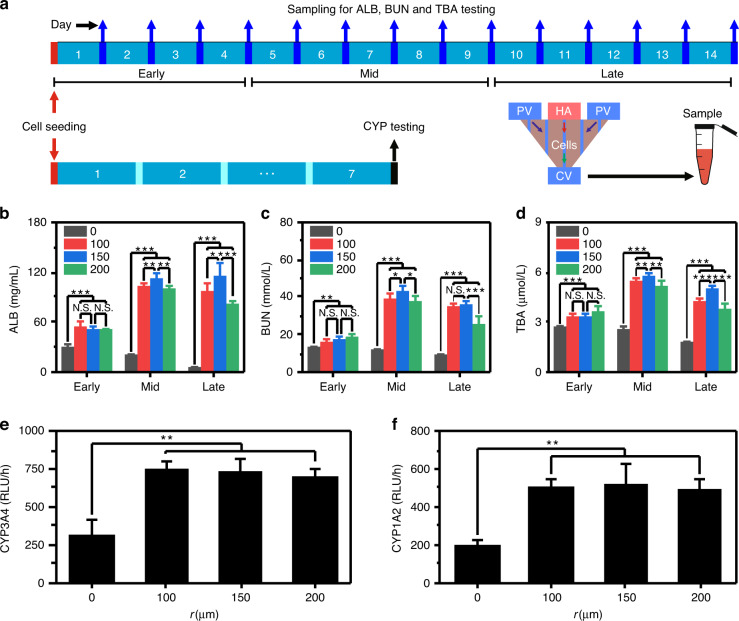
Metabolic analysis of hepatocytes. **a** Schematic of sampling times for metabolic analysis. The average metabolic concentrations of albumin (ALB; **b**), blood urea nitrogen (BUN; **c**) and total bile acids (TBA; **d**) in the early, mid, and late stages of culture. The activities of CYP3A4 (**e**) and CYP1A2 (**f**) on Day 7

To demonstrate the advantages of microneedle-assisted constructed sinusoids in terms of the metabolic function of hepatocytes, the concentrations of specific biomarkers were analyzed (Fig. 4a). For the uninduced group, the concentrations of the studied biomarkers decreased rapidly after a small rise at the beginning of culture (Fig. S7; the daily metabolism also indirectly confirms the stability of the microsystem). However, for the induced groups, the concentrations of the studied biomarkers maintained an upward trend, then reached a maximum at the end of the middle stage, and finally decreased slightly (although the concentrations dropped slightly, the values were still high). Collectively, compared with the microneedle-unused group (i.e., *r* = 0 μm), the concentrations of ALB (Fig. 4b), BUN (Fig. 4c), and TBA (Fig. 4d) in the microneedle-assisted groups were significantly higher, whether in the early stage (1–4 days), in the mid-stage (5–9 days), or in the late stage (10–14 days). In addition, the group with 150 μm microneedles is significantly better than the other groups with microneedles, probably profiting from the more physiologically similar flows provided by sinusoids formed with 150 μm microneedles. We further analyzed the activities of 2 cytochrome P450 enzymes, CYP3A4 (Fig. 4e) and CYP1A2 (Fig. 4f), on Day 7. The results show that the activities of 2 enzymes in the microneedle-assisted groups are also significantly higher than in the microneedle-unused group.

### Microneedle-assisted liver chip increases the viability of cells and promotes the development of microstructures

**Fig. 5:**
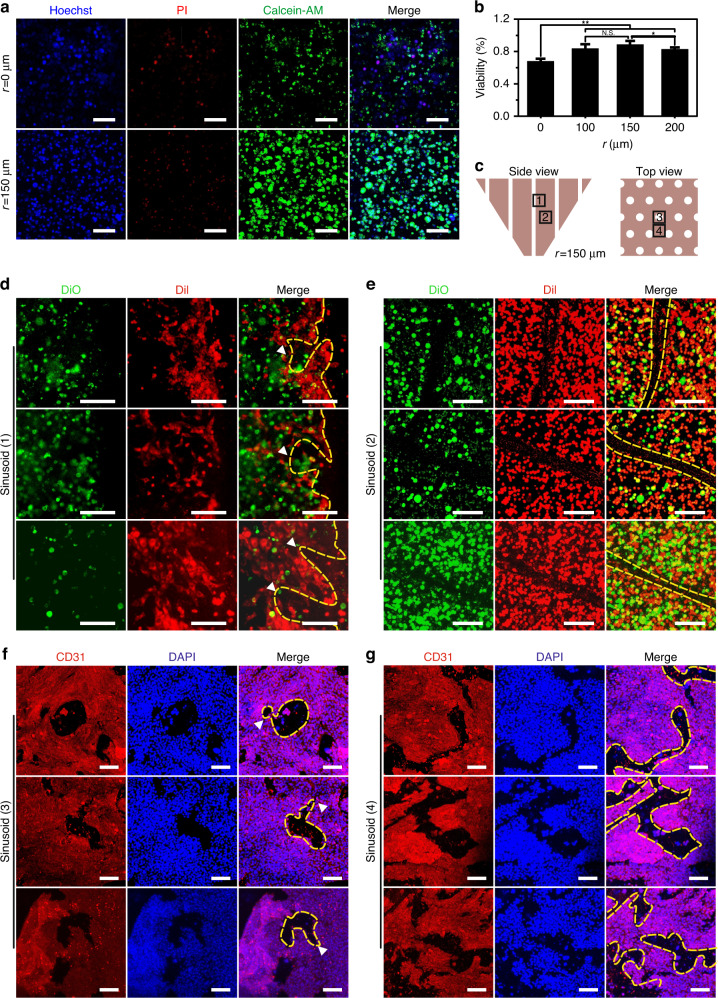
Cell viability and formed liver microstructure. **a** Staining of living and dead cells and **b** statistical results of cell viability. White scale bar = 100 μm. **c** Schematic of the specific staining observation position, wherein 1 and 2 side views and 3 and 4 are top views. DiI (HepaRG) and DiO (human hepatic sinusoid endothelial cell, HHSEC) staining of primary sinusoids formed with a microneedle array at position 1 (**d**) and position 2 (**e**). The yellow dashed line represents the boundary of the primary sinusoid, and the white arrows point to the secondary sinusoid. White scale bar = 150 μm. Specific staining of HHSEC at position 3 (**f**) and position 4 (**g**). The yellow dashed line represents the boundary of the primary sinusoid, and the white arrows point to the secondary sinusoid. White scale bar = 150 μm

To demonstrate the effects of induced sinusoids on liver tissue formation, the viability of cells and the formed microstructures were examined. The induced group presented higher cell viability than the uninduced group (Fig. 5a, b and Fig. S8). Among the induced groups, the viability of the group with a radius of 150 μm microneedles was the highest. In the induced group with 150 μm radius microneedles, the sinusoids formed with microneedles were clearly visible and could be observed more at different positions in the tissue (Fig. 5c shows the checked position; Fig. 5d–g); more interestingly, secondary sinusoid formation occurred, resulting from flow-driven angiogenesis across the primary sinusoids (the formed secondary hepatic sinusoids can further deliver nutrients to the cells far away from the primary hepatic sinusoids). In addition, staining for the enzyme CYP and the bile canaliculus showed the cord-like endothelial structure, the plate-like hepatocyte cluster, and occasionally the bile canaliculus (Fig. S8), which also confirmed the previously tested metabolic results (Fig. 4).

### Reconstruction of concentration gradients and chip application

**Fig. 6:**
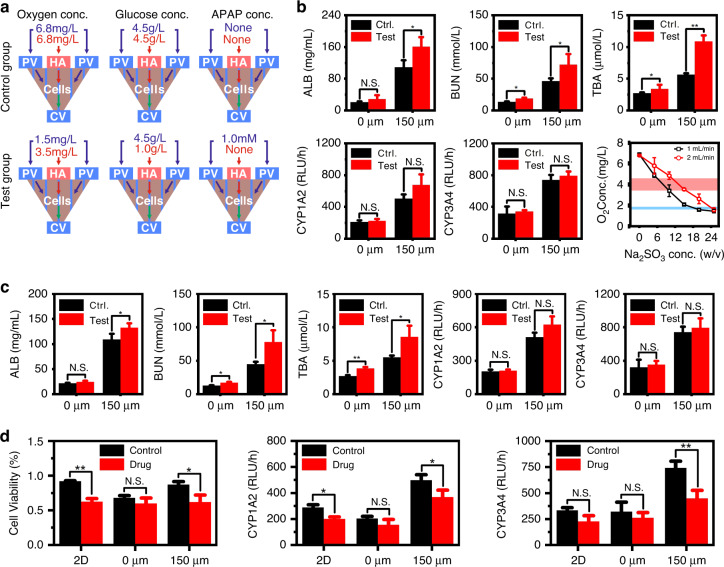
Effects of reconstructed oxygen and glucose gradients on cell metabolism and the application of the chip for drug testing. **a** Schematic of culture conditions for studies of oxygen gradient, glucose gradient, and drug testing. Here, the test drug was acetaminophen. **b** Results of the metabolic difference after oxygen concentration gradient culture and the oxygen concentration control ability of our device. The light red and light blue areas indicate the dissolved oxygen concentrations of HA and PV in the human body in the literature^89^, respectively. **c** Results of the metabolic difference after glucose concentration gradient culture. **d** Results of the cell viability and CYP metabolic difference after drug testing

To demonstrate the physiological similarity of the chip, the effects of the reconstructed oxygen and glucose gradients on cell functions were preliminarily presented (Fig. 6a and Fig. S9). Previous studies have shown that oxygen gradients promote the emergence of hepatocyte diversity and enhance hepatocyte metabolism^48,56,65^. Here, the reconstructed oxygen gradients (simulated in Fig. S10A) also significantly increased the metabolism of hepatocytes as measured by ALB, BUN, TAB, CYP1A2, and CYP3A4 (Fig. 6b). We also checked the influence of glucose gradients (simulated in Fig. S10B) on cell metabolism. One of the key functions of hepatocytes is their response to glucose gradients and their subsequent launch of differentiated metabolism^66,67^. The experimental results of this study show that the more physiologically similar the glucose gradients are, the higher the metabolic activity of hepatocytes (Fig. 6c). That is, the formed sinusoids in the induced groups significantly improved the physiological similarity of oxygen and glucose gradients in mHAC and thus increased the metabolic capacity of hepatocytes. When hepatocytes are exposed to more physiologically similar conditions, they are expected to show a more physiologically similar response to the tested drugs^68^. To demonstrate the application of the mHAC in drug testing, the drug acetaminophen (APAP) was used (APAP is a widely used analgetic and antipyretic drug, and the excessive use of APAP can cause hepatotoxicity^48,69^). The results of this study showed that the hepatocytes in the induced groups were more sensitive to drugs than those in the uninduced groups, especially in terms of CYP enzymes (Fig. 6d).

## Discussion

Structure is the basis of function^70–72^. Existing few studies show that the formation of hepatic sinusoids (i.e., liver microvascular networks) is not only of great significance for the formation of the liver and its tissue function^35^ and the expansion of liver-chip applications^68^ but also provides a prerequisite for the high activity of large-scale liver tissue cultured in vitro^73^. However, the formation of hepatic sinusoids has always been a challenge for liver chips. This is because the size of hepatic sinusoids is very small (only a few microns^74^). This accuracy issue exists for most organ-on-a-chip techniques, even 3D bioprinting^75^. Induced self-assembly by flow, as well as chemical factors, is promising^48,68^, but the few methods proposed by previously reported work have not reproduced the in vivo structural features well^34^. In this paper, a method is proposed for the construction of hepatic sinusoids. That is, a 3D-printed microneedle array is used as a mold; larger primary hepatic sinusoids are formed by demolding technology, and then smaller secondary hepatic sinusoids are self-assembled with the help of microflows. This work clearly shows that primary hepatic sinusoids are formed, followed gradually by secondary hepatic sinusoids. In addition, the fluid flow supplied by the microsystem could exert a positive effect on the viability and function of hepatocytes in long-term culture, as reported previously^48^. Due to the limitations of the printing technique, the microneedles used in this paper are still large (on the order of 100 microns), but the proposed method has the potential to further optimize the formation of the primary hepatic sinusoids by controlling the size, density, and array of the microneedles.

Here, the liver chip is designed based on the physiological hepatic acinus structure, which solves the difficulty of designing a dual blood supply liver chip with a tri-vascular structure. Benefiting from the realization of dual blood supply (PV and HA supply blood separately) and the assistance of the oxygen chip, the liver-chip microsystem can reproduce oxygen zonation well^48^, thus providing a basis for further improvement of liver function. The liver chip can also be used to simulate the dynamic blood glucose distribution^1^ or drug distribution^76^ in a hepatic lobule. In this study, as an example, only one triangular prism was designed on the mHAC; however, more triangular prisms can be carved on the mHAC to obtain a larger-scale liver acinus chip (Fig. S11). In addition, due to the limitation of the chip size, it is very difficult to directly observe the gradient distribution on the chip, so current research on the influence of the gradient is focused on metabolic analysis. In future work, an in-depth study will be performed to fully demonstrate these applications using the liver chip we designed. In addition, the liver acinus-based design with PV, HA, and CV could also provide a reference for the construction of a more biomimetic liver^77^.

## Conclusions

In summary, we proposed a new method for the construction of hepatic sinusoids in a liver-acinus-on-a-chip microsystem using a self-developed microneedle array. We confirmed the formation of hepatic sinusoids and explored the effects of microneedle sizes. Additionally, we preliminarily demonstrated the effects of the resulting oxygen and glucose gradients on hepatocyte functions and the application of the microsystem in drug testing. In the future, liver organoids will be seeded in the cell culture area to further enhance the structure and functional formation of hepatic sinusoids.

## Materials and methods

### Cell culture

Undifferentiated HepaRG cells were obtained from Beina Chuanglian Biotechnology Co., Ltd. (Beijing, China). To obtain differentiated HepaRG cells, undifferentiated HepaRG cells were cultured in William’s E medium (Thermo Fisher Scientific, MA, USA) supplemented with 10% fetal bovine serum (Life Science Products & Services, Australia), 1% penicillin–streptomycin (Sangon Biotech Co., Ltd., Shanghai, China), 2 mM glutamine (Thermo Fisher Scientific, MA, USA), 5 µg/mL insulin (Wanbang Pharmaceutical Co., Ltd., Jiangsu, China) and 50 µM hydrocortisone hemisuccinate (Aladdin Industrial Corporation, Shanghai, China) in a cell incubator (Thermo Fisher Scientific, Waltham, MA, USA) with 5% CO_2_ at 37 °C for 2 weeks and then transferred to the above culture medium containing 2% dimethyl sulfoxide (Sinopharm Chemical Reagent Co., Ltd., Shanghai, China) for 2 weeks^78^. HHSECs were obtained from Tongpai Biotechnology Co., Ltd. (Shanghai, China) and maintained in Dulbecco’s modified Eagle’s medium (DMEM) (Gibco Life Technologies, USA) supplemented with 10% fetal bovine serum, 1% penicillin–streptomycin and 2 mM glutamine in an incubator at 37 °C with 5% CO_2_.

### Hepatic acinus chip fabrication

Here, the hepatic acinus chip was made of three PMMA (Qingsheng Information Technology Co., Ltd., Hefei, China) plates and two SMs (Jin Yi Hong Plastic Materials Co., Hefei, China). The chip was designed with SolidWorks software (Fig. S1) and fabricated using a CNC drill/tap center (DT-1, Haas Automation, Inc., CA, USA) at the Experiment Center for Life Sciences at the University of Science and Technology of China (USTC), Anhui, China (Fig. S2).

### Microneedle array and auxiliary mold fabrication

The microneedle array and auxiliary mold for demolding were designed with SolidWorks and fabricated using a high-precision 3D printer (S130, 2 μm precision, BMF Material Technology Inc.) at the Engineering and Materials Experiment Center in USTC, Anhui, China (Fig. S3). Here, three microneedle arrays were designed and fabricated, and the ratio of microneedle space (s) to microneedle diameter (2*r*) was 5:2 (Fig. 1C). Due to its excellent photocuring properties and biocompatibility, GelMA was adopted as the ECM^79,80^. After the microneedle array was removed from the GelMA hydrogel, the cylindrical channel arrays modeling hepatic sinusoids formed in the hydrogel. In this study, the porosity (*ε*) of the liver tissue is defined as the volume ratio of the hepatic sinusoids to the liver tissue in the following equation:

$${\rm{\varepsilon }}=\frac{{V}_{{sinusoid}}}{{V}_{{tissue}}}=\frac{\pi {r}^{2}}{\frac{\sqrt{3}}{2}{s}^{2}}$$

the value of *ε* can be calculated as 0.145, which is in the range (0.143 ± 0.028) of the porosity of human liver tissue reported in the literature^81^. The microneedle space of the through-holes in the auxiliary mold is the same as that of the microneedle array, and the radius of the through-holes in the auxiliary mold is 200 μm larger than that of the microneedle array.

### Cell loading and chip assembly

Before chip assembly, the GelMA precursor solution was prepared according to the supplier’s instructions. Briefly, GelMA solid was dissolved in phosphate-buffered saline (PBS) containing 0.25% photosensitizer at 60 °C for 30 min to obtain the GelMA precursor solution (w/v: 10%). The solution was sterilized using a filter (pore size: 0.22 μm) before use. For chip assembly, Layer 2 of the mHAC, microneedle array, and auxiliary mold were assembled in the order shown in Fig. 1d. The differentiated HepaRG cells and HHSECs were mixed with GelMA precursor solution (the cell densities were approximately 2 × 10^7^ cells/mL and 4 × 10^6^ cells/mL, respectively), and the cell-laden GelMA solution was injected into the culture area in layer 2 and irradiated with 405 nm ultraviolet (UV) light for 30 s (short-term UV illumination has very little effect on cell viability^82–84^). Afterward, the auxiliary mold and microneedle array was carefully demolded in sequence (it should be noted that due to mold assembly error, blocking of primary sinusoids sometimes occurred after demolding, especially at the bottom; thus, a slightly longer microneedle array is proposed; moreover, the flow shear force may also alleviate the blocking). Then, Layers 1, 2, and 3 of the mHAC, SMs, and porous membranes (10 mm × 5 mm × 0.1 mm) were assembled in that order (Fig. 1e). Finally, the cell suspension of HHSECs (the cell density was approximately 4 × 10^6^ cells/mL) was injected into the chip from PV and HA inlets, and the chip was cultured in an incubator at 37 °C with 5% CO_2_ overnight to allow cell attachment (Fig. 1f).

### Oxygen concentration regulating chip fabrication

The oxygen concentration regulating chip (ORC) contains two PMMA plates and one SM layer. The detailed fabrication parameters are given in Fig. S12. Compared to the oxygen chip we previously reported^48^, the stability of the ORC reported here was enhanced, especially under high flow rates, by increasing the width of the chip.

### Assembly and operation of the liver-chip microsystem

The mHAC, ORC, glass flasks, silicone tubes, and PTFE tubes were connected as shown in Fig. 1f (the picture of the culture system is in Fig. S4). It should be noted that the lengths of the silicone tubes were the same, while the lengths of the PTFE tubes for PV, HA, and CV (wavy lines in Fig. 1f) were 10 cm, 5 cm, and 2 cm, respectively, to promote the generation of interstitial flow. Here, the tape was used to prevent fluid leakage at the connections. The liver chip, the glass flasks, and the oxygen concentration regulating chips were placed in the cell incubator, while the peristaltic pumps were placed outside the incubator. The flow rates of the PV, HA, and CV channels were set to 2, 1, and 1 mL/min, respectively, and operated continuously for 7–14 days.

### Microneedle arrays and sinusoid characterization

In this study, SEM (EVO18, Zeiss, Germany) was used to characterize the morphology of the microneedle array. To enable observation of the nonconductive microneedle array under SEM, the microneedle array was gilded by a vacuum evaporation instrument. Then, the gilded microneedle array was observed and photographed by SEM. We measured the bottom circle area of the cylindrical microneedles and calculated the radius using ImageJ software.

CLSM (DMi8, Leica, Germany) was used to characterize the GelMA hydrogel, and the three-dimensional structure was reconstructed. To observe the 3D structure of ECM, GelMA labeled with blue fluorescence was used (the operation procedure in the section “*cell loading and chip assembly”*). The drilling GelMA hydrogel was removed from the chip with a scalpel and immediately photographed using CLSM at different resolutions. For the measurement of the area and radius of the bottom circle of the cylindrical channel, ImageJ software was used.

### Flows inside ECM analysis

In this study, we analyzed the flow and direction of interstitial flow. The uninduced cell-free tri-vascular system and the induced systems (induced by different microneedle arrays with *r* = 100 μm, 150 μm, and 200 μm) were constructed as described in the section “*assembly and operation of the liver-chip microsystem”*. We used COMSOL to simulate the flow in the tissue, and more detailed information can be found in Fig. S5. Based on the simulation results, the flow rates of the PV, HA, and CV inlets were set to 2, 1, and 1 mL/min, respectively, the outlet pressure difference was 8 Pa, and the system was operated for 1 h. After the system was stable, the liquid from the CV outlet within 5 minutes was collected. In this study, interstitial flow is defined as the flow from HA and PV to CV, which was calculated using the following equations:

$${Q}_{{CO}}-{Q}_{{CI}}={Q}_{{PO}}-{Q}_{{PI}}+{Q}_{{HO}}-{Q}_{{HI}}={Q}_{t}$$

where ${Q}_{{CO}}$, ${Q}_{{PO}}$ and ${Q}_{{HO}}$ are the outlet flow rates of CV, PV, and HA, respectively. ${Q}_{{CI}}$, ${Q}_{{PI}}$, and ${Q}_{{HI}}$ are the inlet flow rates of CV, PV, and HA, respectively, and ${Q}_{t}$ is the interstitial flow.

To confirm the flow direction on the chip, deionized water with blue dye was used. We first measured the absorbance of water with blue dye (0.12 mg/mL) in the wavelength range of 400–800 nm and found that the dye had an absorption peak at 622 nm. As the dye concentration ranged from 0 to 0.3 mg/mL, there was a linear correlation between the dye concentration and the dye absorbance at 622 nm, as shown in Fig. S13. Thus, deionized water with the dye added at a concentration of 0.3 mg/mL was pumped into the mHAC through the PV, HA, or CV inlet, and the absorbance of the water with dye from all the inlets and outlets of the mHAC at 622 nm was recorded using a microplate reader (Multiskan GO; Thermo Fisher Scientific, MA, USA).

### Hepatocyte metabolism analysis

To analyze liver function, the concentrations of TBA, ALB, and BUN metabolized by the HepaRG cells were tested, as well as the concentrations of CYP1A2 and CYP3A4 enzymes. To estimate the TBA, ALB, and BUN concentrations, medium samples collected from the medium bottle connected to the CV outlet of the chip were analyzed with an enzyme-labeled reader using a human ELISA kit (Mlbio, Shanghai, China) according to the literature^59,68,85^. For CYP1A2 and CYP3A4, the device was disassembled on the 7th day, and the cultured tissue was placed in a 48-well plate. The CYP1A2 and CYP3A4 activities were measured using P450-Glo™ CYP1A2 and CYP3A4 assays (Promega, Madison, WI, USA) according to the manufacturer’s instructions, and the luminescence was read by a microplate reader (iD5, Molecular Devices, USA)^26,53,86,87^.

### Analysis of cell viability

Cell viability was determined by cell staining. The device was disassembled on Day 7, and the cultured tissue was placed in a 48-well plate for cell staining. Calcein-AM (Aladdin Industrial Corporation, Shanghai, China), propidium iodide (PI, Sangon Biotech Co., Ltd., Shanghai, China), and Hoechst 33342 (Sangon Biotech Co., Ltd., Shanghai, China) were used to identify living cells, dead cells, and cell nuclei, respectively (Fig. S14A). The cells were stained according to the manufacturer’s instructions and observed using a fluorescence microscope (IX73; Olympus, Tokyo, Japan)^49^. ImageJ software was used to analyze the viability of cells (i.e., the ratio of the number of living cells to the number of cell nuclei).

### Immunostaining and imaging

After 7 days of culture, the device was disassembled, and the cultured tissue was fixed in 4% paraformaldehyde for 30 min at room temperature and then permeabilized with 0.2% Triton X-100 (Sigma) in PBS solution for 15 min at room temperature. After blocking with 2% bovine serum albumin (BSA, Sigma) in PBS for 1 h at room temperature, samples were incubated overnight at 4 °C with primary antibodies (anti-CD31, anti-ZO-1, anti-CYP1A2, and anti-CYP3A4; 1:100 in PBS; Proteintech Co., Ltd., Hubei, China) and then incubated for 4 h at 4 °C with the corresponding secondary antibodies (Alexa Fluor 488 or 594; 1:250 in PBS; ABclonal Biotech Co., Ltd., Hubei, China). Cell nuclei were stained with 4’,6-diamidino-2-phenylindole (DAPI; 1:100 in PBS; Proteintech Co., Ltd., Hubei, China) for 30 min at room temperature. The samples were washed 3 times with PBS after each step (Fig. S14B and Fig. S14C). Images of the samples were taken using CLSM.

### Bile canaliculi staining

The successful formation of bile canaliculi was verified using 5(6)-carboxy-2’,7’-dichlorofluorescein diacetate (CDFDA, Sigma‒Aldrich, MO, USA)^88^. After staining with CDFDA (1:300 in PBS dilution) for 30 min at 37 °C, the cells were washed 3 times with PBS and then observed using a fluorescence microscope (Fig. S14D).

### Prestaining

Before cell loading and device assembly, cells were stained with DiI (red, 1:100 in PBS) or DiO (green, 1:100 in PBS) (Jiangsu Keygen Biotechnology Co., Ltd., Jiangsu, China) dye at 37 °C for 30 min, followed by washing 3 times with PBS (Fig. S14E).

### Oxygen gradient culture

To create an oxygen gradient in the chip, the ORC was connected to the liver chip to generate an oxygen gradient in the tissue area. According to the literature, the dissolved oxygen concentrations for HA and PV were maintained at 3.5 and 1.5 mg/L, respectively^48^. The device was run for 7 days in an incubator at 37 °C with 5% CO_2_ (Fig. S9A).

### Glucose gradient culture

To develop a glucose gradient in the chip, the PV, HA, and CV bottles were loaded with high-glucose DMEM (4.5 g/L, Gibco Life Technologies, USA), low glucose DMEM (1.0 g/L, Gibco Life Technologies, USA) and low-glucose DMEM (1.0 g/L), respectively. The cell medium in each bottle was supplemented with 10% fetal bovine serum, 1% penicillin–streptomycin, and 2 mM glutamine. The device was run for 7 days in an incubator at 37 °C with 5% CO_2_ (Fig. S9B).

### Drug test

Drug testing of the liver chip was demonstrated (Fig. S9C). The chip was cultured for 6 days. For the microneedle-induced group (*r* = 150 μm) and the uninduced group, medium mixed with APAP (1 mM in DMEM, as reported in the literature^48^) was pumped into the chip through the PV. For the control group, medium mixed with APAP (1 mM in DMEM) was added to the petri dish. After treatment with the drug for 24 h, the CYP activity and viability of cells in the three groups were tested.

### Statistical analysis

Data of continuous variables are presented as the means ± standard deviations of triplicate experimental results. *p* Values were analyzed using a two-tailed Student’s *t*-test or one-way ANOVA. *p* Values < 0.05 were considered significant. The significance levels are indicated by * for *p* < 0.05, ** for *p* < 0.01, *** for *p* < 0.001, and N.S. for no significance.

## Supplementary information

Supplementary Information
