# Supplementary material for: Microneedle array facilitates hepatic sinusoid construction in a large-scale liver-acinus-chip microsystem
Source: Microsyst Nanoeng. 2023 Jun 7;9:75. doi: 10.1038/s41378-023-00544-w (PMC10247758; doi:10.1038/s41378-023-00544-w)
Supplement: Supplementary file 1 — Supplementary Information [file 41378_2023_544_MOESM1_ESM.pdf]

Supplementary Information

Microneedle array facilitates hepatic sinusoid construction in a large-scale liver-acinus-chip microsystem

*Shibo Li [a, b], Chengpan Li [a], Muhammad Imran Khan [c], Jing Liu [d], Zhengdi Shi [a], Dayong Gao [e], Bensheng Qiu [c], Weiping Ding [b, *]*

[a] Department of Electronic Engineering and Information Science, University of Science and Technology of China, Hefei, Anhui 230027, China

[b] Department of Oncology, the First Affiliated Hospital of USTC, Division of Life Sciences and Medicine, University of Science and Technology of China, Hefei, Anhui 230001, China

[c] Center for Biomedical Imaging, University of Science and Technology of China, Hefei, Anhui 230027, China

[d] School of Biology, Food and Environment, Hefei University, Hefei, Anhui 230601, China

[e] Department of Mechanical Engineering, University of Washington, Seattle, WA 98195, USA

*Corresponding author:

Weiping Ding, E-mail: wpdings@ustc.edu.cn; ORCID: 0000-0002-3331-1011

S1. Design parameters of the microneedle-assisted hepatic acinus chip (mHAC)

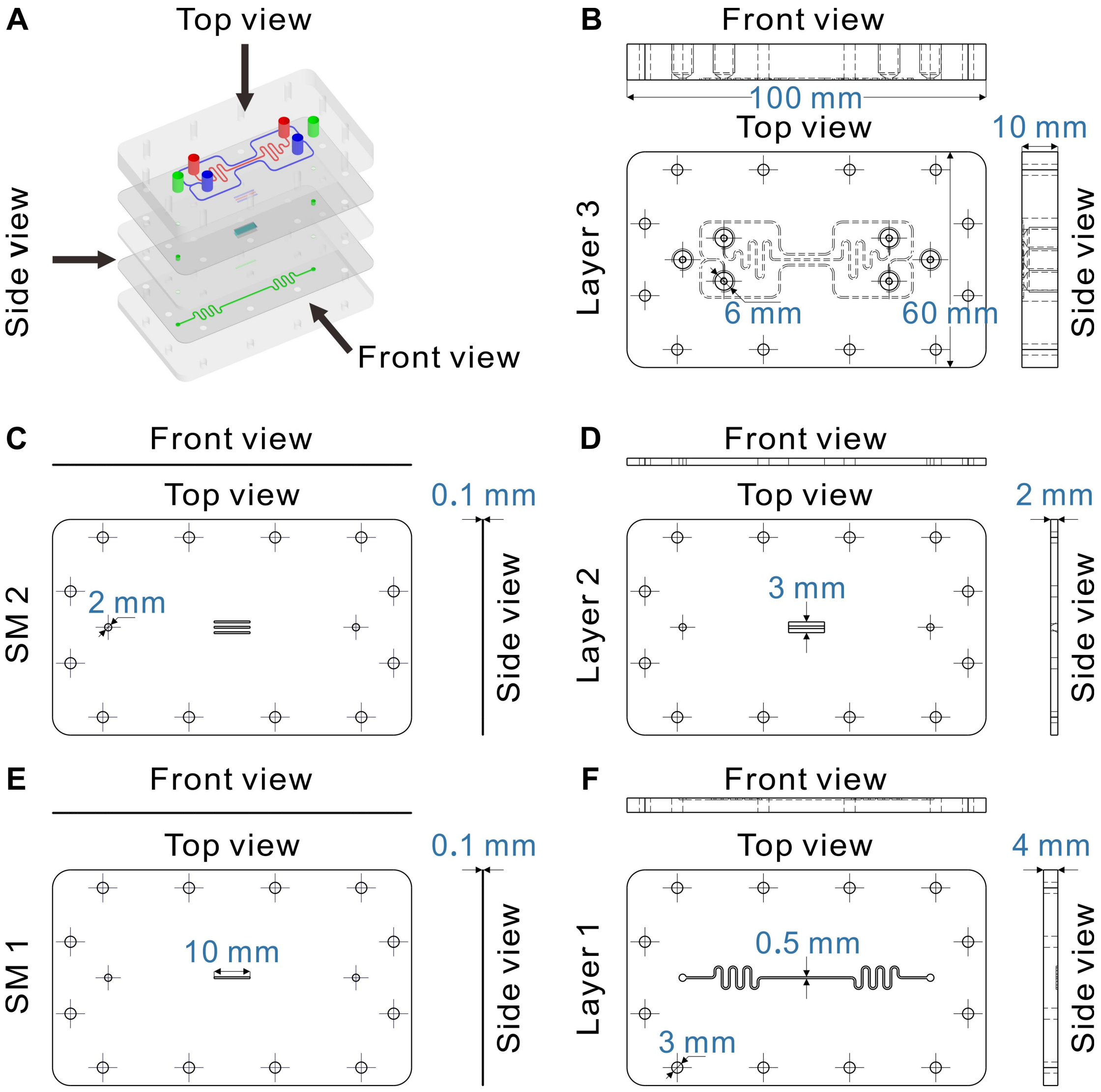

Figure S1. Design parameters of the mHAC. (**A**) Schematic of the components of mHAC. (**B-F**) Design parameters of Layer 3, SM 2, Layer 2, SM 1 and Layer 1.

S2. Pictures of the mHAC

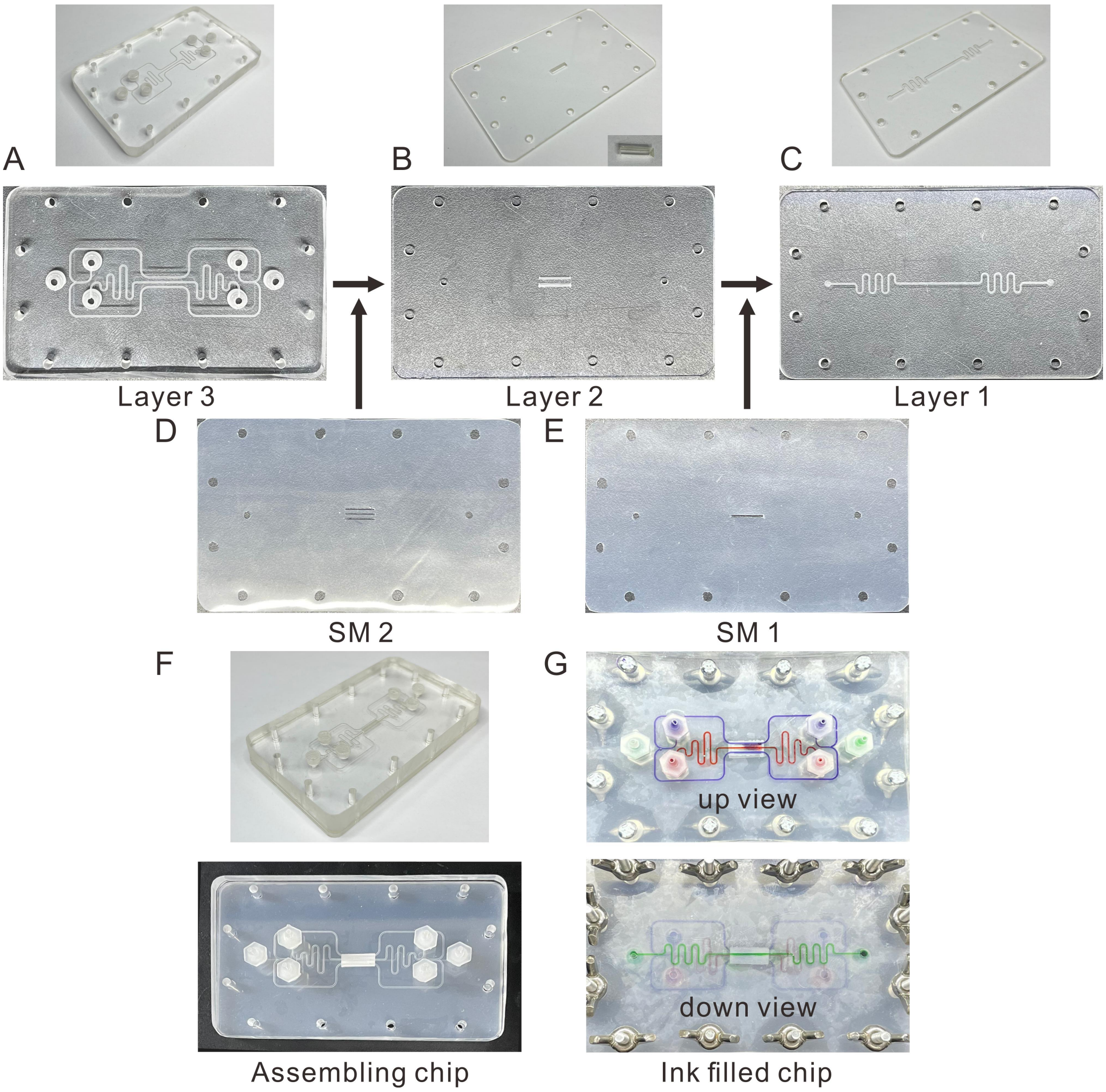

Figure S2. Pictures of the mHAC. Subgraphs (**A-E**) show pictures of (**A**) Layer 3, (**B**) Layer 2, (**C**) Layer 1, (**D**) SM 2 and (**E**) SM 1. Subgraph (**F**) shows the assembly of mHAC. Subgraph (**G**) shows the ink-indicated flows in CV (green), PV (blue) and HA (red).

S3. Design parameters of the microneedle array and its auxiliary mold

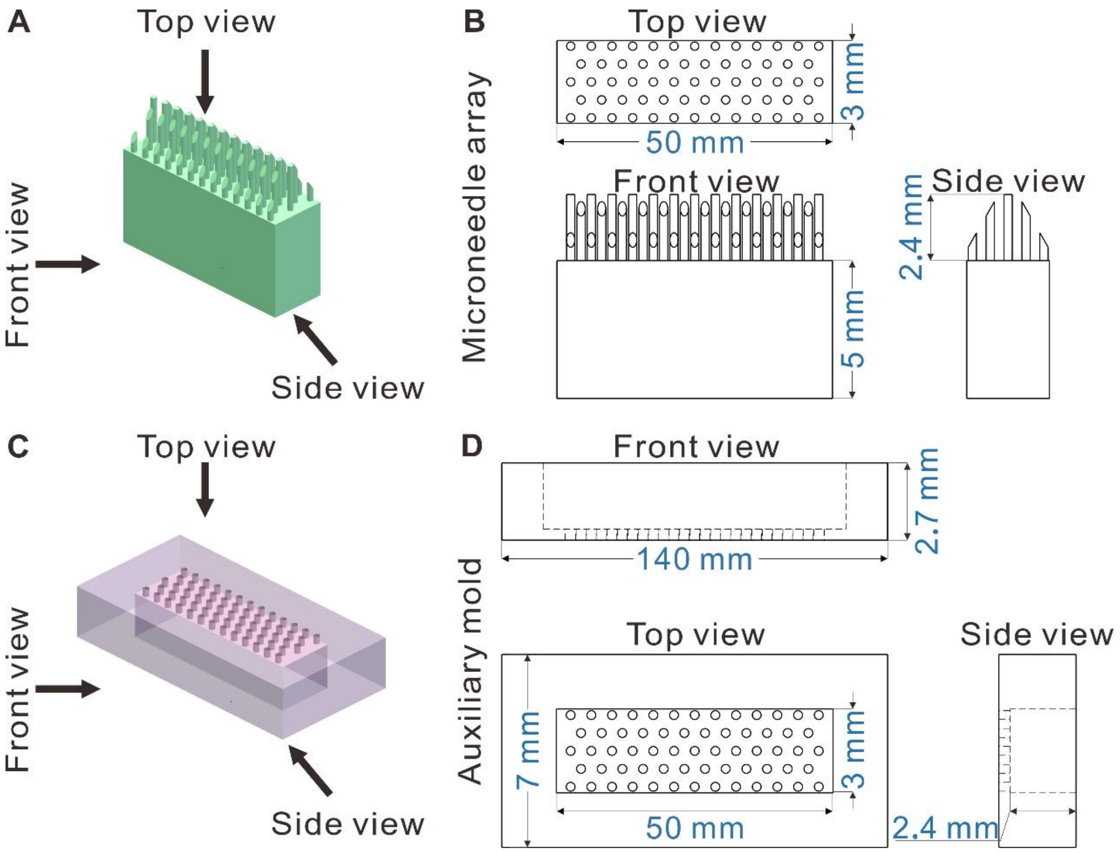

Figure S3. Design parameters of the microneedle array and its auxiliary mold. Here, a microneedle with a radius of 150 μm is shown as an example. (**A**) Schematic of the microneedle array. (**B**) Design parameters of the microneedle array. (**C**) Schematic of the auxiliary mold. (**D**) Design parameters of the auxiliary mold.

S4. Photograph of the assembled liver-acinus-chip culture system

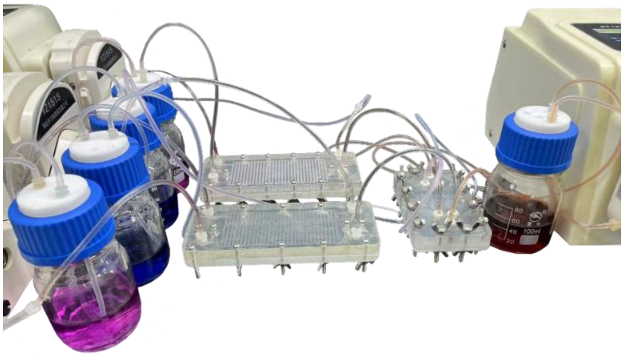

Figure S4. Photograph of the assembled liver-acinus-chip culture system.

S5. Flow simulation

In this study, the software COMSOL was used to simulate the flow in the cell culture area. The assumptions for modeling are as follows: (1) the fluid is incompressible; (2) the flow is laminar; (3) the basic properties of the culture medium (density, viscosity, etc.) are the same as water at 37 °C; (4) both the porous membrane and ECM are homogeneous porous media.

Following the above-mentioned assumptions, the free and porous media flow module was used to calculate the flows in the regions of pores (sinusoids) and tri-vascular pathways, in the porous membrane region, and in the region of the cell-loaded ECM area [1-5]:

Region 1: Pore/PV/CV/HA

$$\rho\nabla\bullet\mathbf{u_1}=0$$
$$\rho\left[\left(\mathbf{u_1}\bullet\nabla\mathbf{u_1}\right)\right]=\nabla\bullet\left[-p_1\mathbf{I}+\mu\left(\nabla\mathbf{u_1}+\left(\nabla\mathbf{u_1}\right)^T\right)\right] \tag{1}$$

Region 2: Porous Membrane

$$\rho\nabla\bullet\mathbf{u_2}=0$$
$$\frac{\rho}{\varepsilon_2}\left[\left(\mathbf{u_2}\bullet\nabla\right)\frac{\mathbf{u_2}}{\varepsilon_2}\right]=\nabla\bullet\left[-p_2\mathbf{I}+\frac{\mu_2}{\varepsilon_2}\left(\nabla\mathbf{u_2}+\left(\nabla\mathbf{u_2}\right)^T\right)\right]-\frac{\mu_2}{k_2}\mathbf{u_2} \tag{2}$$

Region 3: Cell-Load ECM

$$\rho\nabla\bullet\mathbf{u}_3=0$$
$$\frac{\rho}{\varepsilon_3}\left[\left(\mathbf{u}_3\bullet\nabla\right)\frac{\mathbf{u}_3}{\varepsilon_3}\right]=\nabla\bullet\left[-p_3\mathbf{I}+\frac{\mu_3}{\varepsilon_3}\left(\nabla\mathbf{u}_3+\left(\nabla\mathbf{u}_3\right)^T\right)\right]-\frac{\mu_3}{k_3}\mathbf{u}_3 \tag{3}$$

where, the subscripts 1 2 and 3 denote three regions, respectively; ρ is the fluid density; μ is the fluid viscosity; ε is the porosity; k is the Darcy permeability; $\mathbf{u}$ is the fluid velocity vector; $\mathbf{I}$ is the unit tensor; p is the pressure; ∇ is the del operator; and the superscript T is the matrix transposition. The values of the parameters are shown in **Table S1**.

Table S1. Parameters used in flow simulation

Parameters	Values	Units	References
ρ	0.994	g/cm^3	water at 37 °C
μ_2	0.695×10^{-3}	Pa·s	water at 37 °C
μ_3	0.695×10^{-3}	Pa·s	water at 37 °C
μ_{vivo}	4.3×10^{-3}	Pa·s	6
ε_2	0.75		7
ε_3	0.74		8
k_2	1.36×10^{-8}	m^2	9

k_3	1.5×10^{-13}	m^2	10
k_{vivo}	3.3×10^{-13}	m^2	11
Δp_{vivo}	20	Pa	6

In the simulation, the inlet flow rates of HA, PV and CV were 1, 2 and 1 ml/min, respectively and the outlet pressures of PV and HA were the same (the difference between the PV/HA outlet pressure and the CV outlet pressure is defined as Δp). Simulations show that the Δp significantly affects the flow rate (**Figure S5**). Thus, to obtain a physiological flow rate, it is necessary to choose an appropriate Δp. Since the permeability of the ECM used and the viscosity of the medium used in the experiments deviate from the values *in vivo*, the Δp was adjusted according to Darcy's law [4]:

$$\Delta p = \frac{k_{vivo}\mu_3}{k_3\mu_{vivo}}\Delta p_{vivo} \quad (4)$$

where Δp_{vivo}, k_{vivo}, and μ_{vivo} are the pressure difference, tissue permeability and fluid viscosity *in vivo*, respectively. Based on the above-mentioned equation, the Δp is approximately 8 Pa. Under the condition, the flow rate in the group with 150 of microneedles is close to the physiological flow rate [12-17].

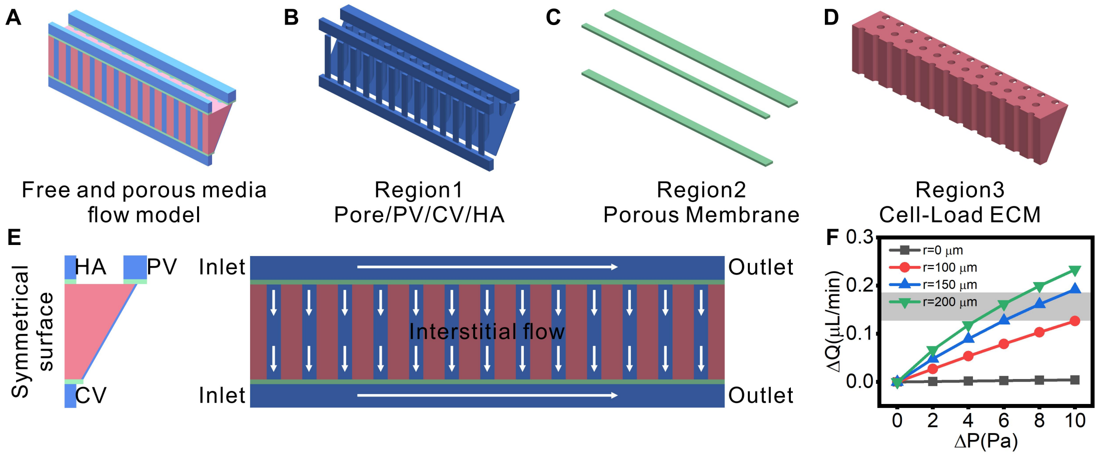

Figure S5. Flow simulation. Subgraphs (**A-D**) show (**A**) the entire simulation area, (**B**) the pore/PV/CV/HA region, (**C**) the porous membrane region, and (**D**) the cell-load ECM region, respectively. (**E**) Schematic of flow conditions. (**F**) The relation between the pressure difference and the permeated flux. The gray area shows the physiological value range [12-17].

S6. Flow direction inside ECM

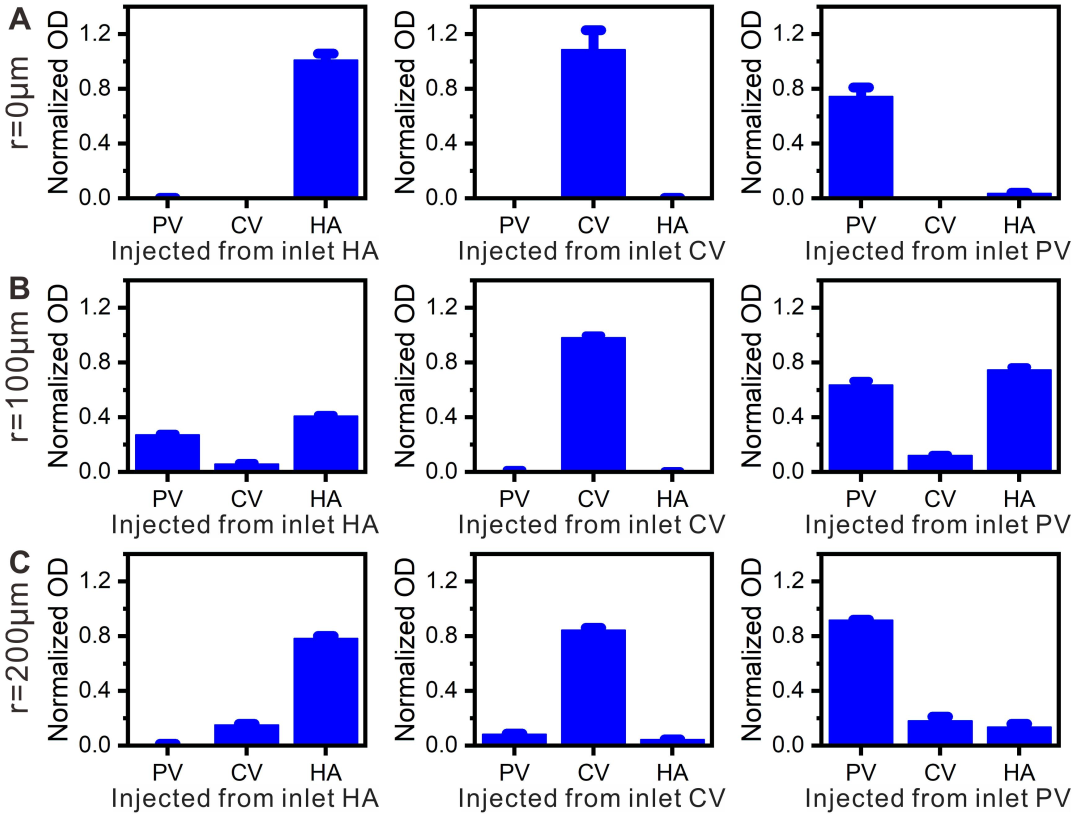

Figure S6. Flow directions inside ECM. (**A**) Sinusoids formed with 0 μm of microneedles. (**B**) Sinusoids formed 100 μm of microneedles. (**C**) Sinusoids formed 200 μm of microneedles. Concentrations of the dye in HA, PV and CV are depicted as the absorbance OD values of the dye.

S7. Daily metabolism

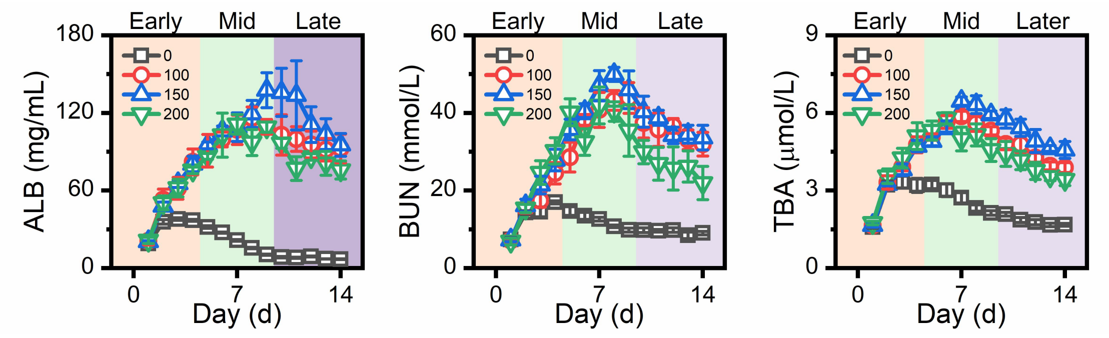

Figure S7. Daily metabolism. Early Stage: from day 1 to day 4. Mid Stage: from day 5 to day 9. Late Stage: from day 10 to day 14.

S8. Cell viability and formed liver microstructure

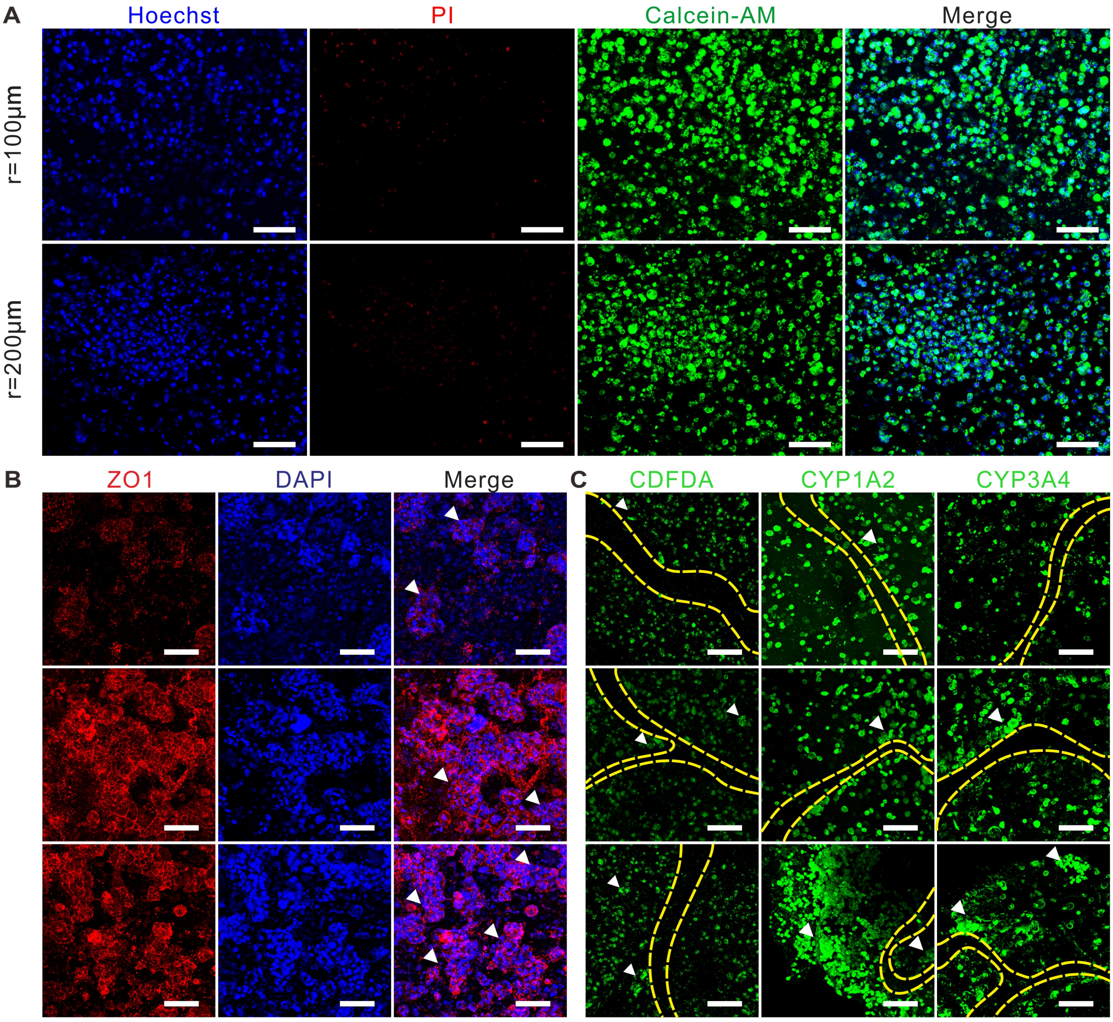

Figure S8. Cell viability and formed liver microstructure. Subgraphs show (**A**) cell viability, (**B**) cell tight junctions, and (**C**) hepatocyte specific staining, bile duct staining. In the figures, white arrows show cell clusters or cord-like structures and the yellow dashed areas show sinusoid-like structures. Scale bar = 100 μm.

S9. Scheme for concentration gradient reconstruction and chip application

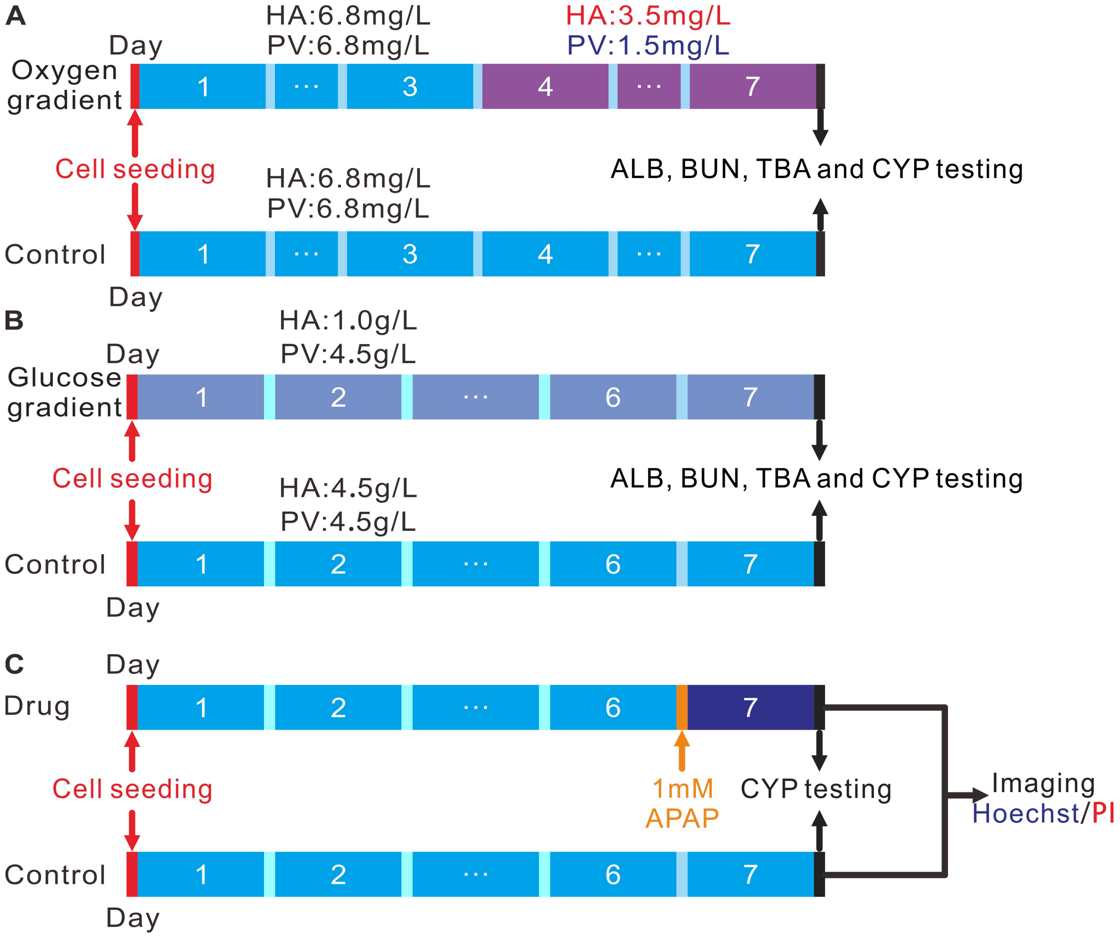

Figure S9. Scheme for concentration gradient reconstruction and chip application. Here, all results were detected after 7 days. (**A**) For the reconstruction of oxygen gradients, in the experimental groups, the dissolved oxygen concentrations at HA and PV were given after three days of culture, according to physiological conditions [18] while in the control groups, the dissolved oxygen concentrations were the same as those in the incubator. The metabolic capacities of ALB, BUN, TBA and CYP were detected after seven days of culture, and hypoxia staining was performed. (**B**) For the reconstruction of glucose gradients, in the experimental groups, the differentiated glucose concentrations at HA (low) and PV (high) were given while in the control groups, the high glucose concentration was given for both HA and PV. The metabolic capacities of ALB, BUN, TBA and CYP were detected after seven days of culture. (**C**) For the drug testing application, the experimental group was given the culture medium mixed with 1 mM APAP after 6 days of culture, while the control group was given only the culture medium. Cell viability and CYP metabolic capacities were tested after 24 hours.

S10. Simulation of oxygen and glucose gradients

To simulate oxygen and glucose gradients in the mHAC, the following main assumptions were used: (1) the effects of the cell metabolism on the concentration fields were negligible; (2) the effects of the mass transfer process on the flow fields were negligible. Then, the mass transfer of oxygen or glucose can be described using the following convection-diffusion equation [5]:

$$\mathbf{u}\cdot\nabla c+\nabla\cdot(-D\nabla c)=0 \tag{5}$$

where c is the concentration, and D is the diffusion coefficient. In the pore/PV/CV/HA regions, D is the free diffusion coefficient D_0. In the porous membrane and tissue regions, the diffusion coefficient can be calculated as follows [19]:

$$D=D_0\varepsilon^2/(2-\varepsilon)^2 \tag{6}$$

Here, the free diffusion coefficients of oxygen and glucose are 9.0×10^{-10} m^2/s and 2.92×10^{-13} m^2/s, respectively [15,16].

In the simulation, the software COMSOL was used and the main concentration parameters at the inlets are shown in **Table S2** (the flow parameters are the same as the ones in S5).

Table S2. Parameters used in concentration simulation

Substances	Inlets	Values	Units
Oxygen	PV	1.5	mg/L
	HA	3.5	mg/L
	CV	6.8	mg/L
Glucose	PV	1.0	g/L
	HA	4.5	g/L
	CV	1.0	g/L

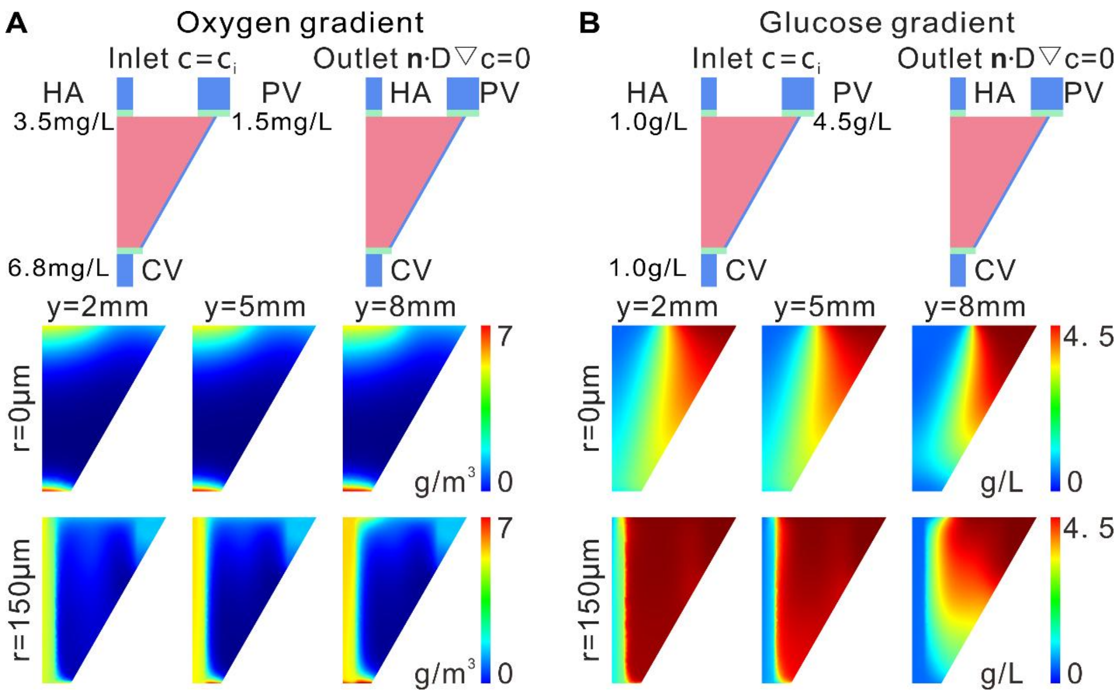

Figure S10. Simulated oxygen and glucose gradients. The concentration settings at the inlets for (**A**) oxygen and (**B**) glucose are shown above the simulated results.

S11. Schematic of the multi-hepatic-acinus chip

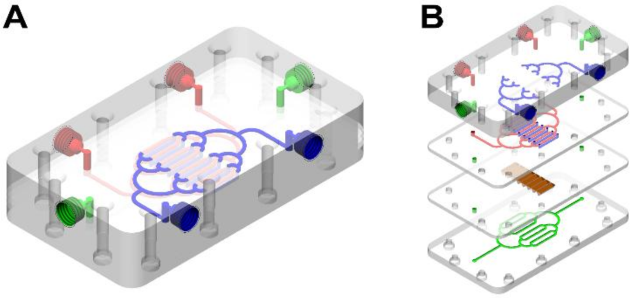

Figure S11. Schematic of the multi-hepatic-acinus chip: assembled chip **(A)** and decomposed chip (**B**).

S12. Design parameters of the oxygen concentration regulating chip

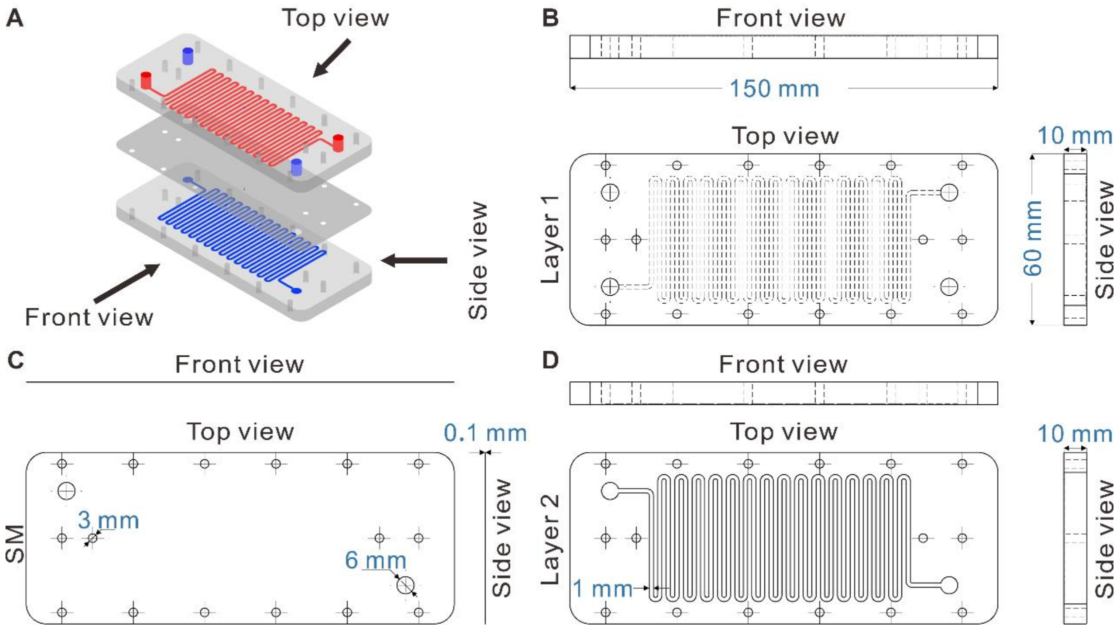

Figure S12. Design parameters of the oxygen concentration regulating chip (ORC). (**A**) Schematic of the components of ORC. (**B-D**) Design parameters of Layer 1, SM, and Layer 2.

S13. Dye absorbance standard curve

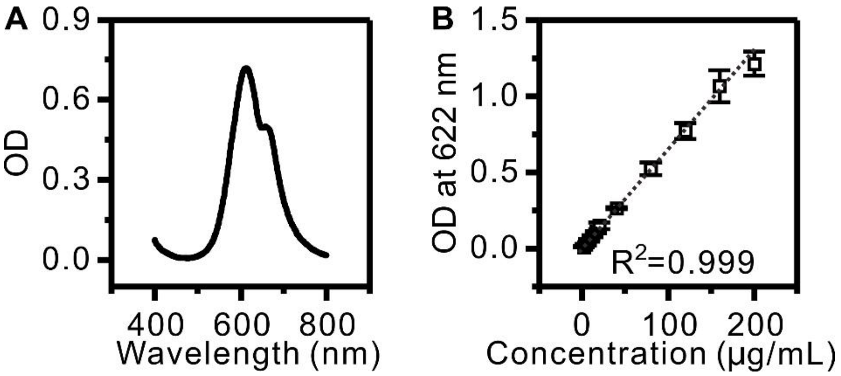

Figure S13. Dye absorbance standard curve. (**A**) Dye absorption spectrum (the dye has an absorption peak at 622 nm). (**B**) Standard curve of the dye absorbance at 622 nm.

S14. Scheme for morphological detection

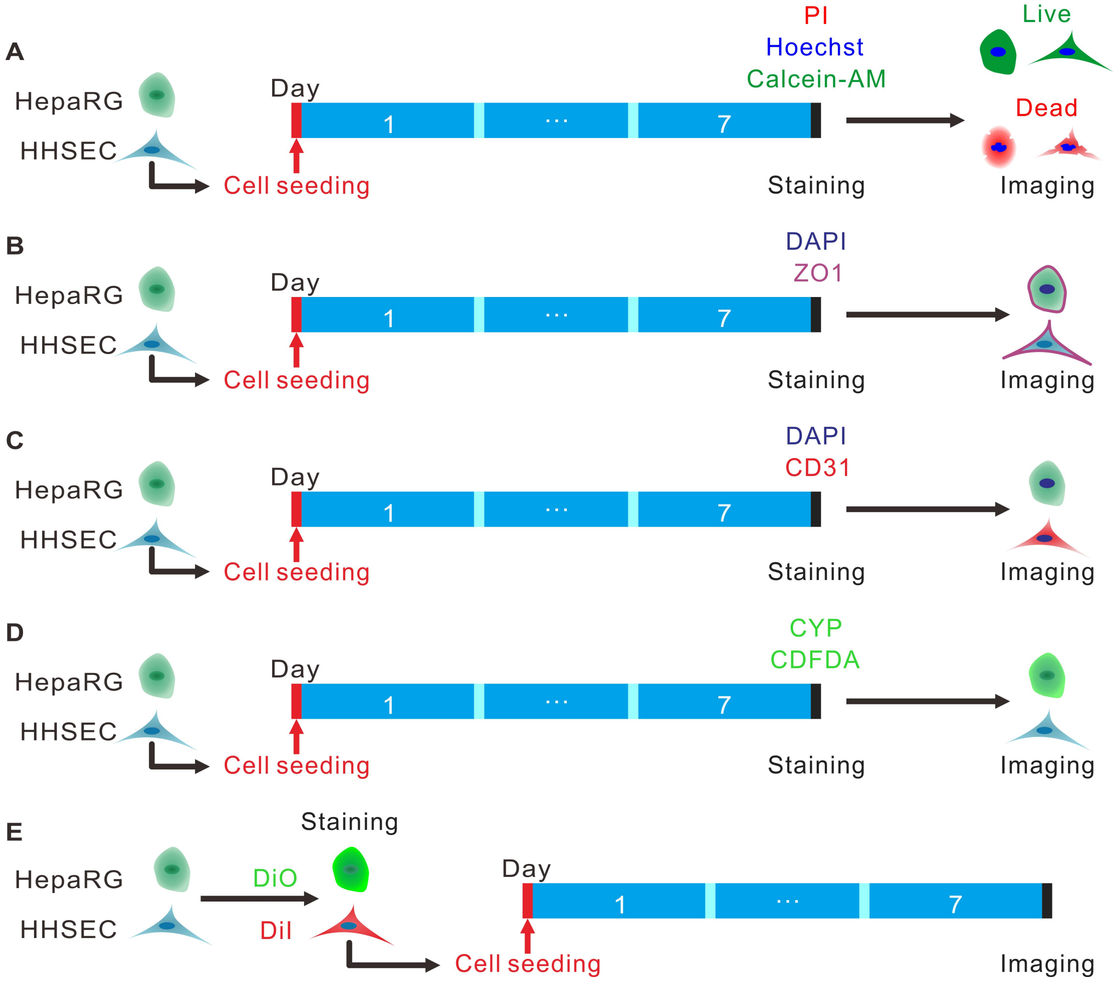

Figure S14. Scheme for microstructure detection. HHSEC and HepaRG cells were directly seeded into the device for 7 days of culture, and then the device was disassembled for staining. (**A**) For viability assays, live cells, dead cells and nuclei were stained using Calcein-AM, PI and Hoechst, respectively. (**B**) For cell tight junction detection, intercellular tight junctions and nuclei were stained using ZO-1 and DAPI, respectively. (**C**) For endothelial cell detection, HHSEC and nuclei were stained using CD31 and DAPI, respectively. (**D**) For hepatocyte function, CYP1A2 and CYP3A4 were stained. For bile canaliculi detection, HepaRG cells were stained with CDFDA. (**E**) For prestaining experiments, HHSEC and HepaRG were pre-stained with Dil and DiO, respectively and then seeded in the device; after 7 days of culture, the device was disassembled for imaging.

References

1. Choi, S. W., Il Lee, W. & Kim, H. S. Analysis of flow characteristics of cryogenic liquid in porous media. *Int. Commun. Heat Mass* **87**, 161-183 (2015).
2. Talebi, F., Mahmoudi, A. H. & Shahi, M. Numerical study of mixed convection flows in a square lid-driven cavity utilizing nanofluid. *Int. Commun. Heat Mass* **37**, 79-90 (2010).
3. Le Bars, M. & Worster, M. G. Interfacial conditions between a pure fluid and a porous medium: implications for binary alloy solidification. *J. Fluid Mech.* **550**, 149-173 (2006).
4. Ding, W. *et al.* Simulation of blood and oxygen distributions in a hepatic lobule with sinusoids obstructed by cancer cells. *J. Theor. Biol.* **446**, 229-237 (2018).
5. Ding, W. *et al.* Estimation of the porosity of a chitosan–carbon nanoparticle membrane fabricated on a chip: a solute transport-based study. *Int. Commun. Heat Mass* **99**, 822-830 (2016).
6. Rani, H. P., Sheu, T. W., Chang, T. M. & Liang, P. C. Numerical investigation of non-Newtonian microcirculatory blood flow in hepatic lobule. *J. Biomech.* **39**, 551-563 (2006).
7. Liu, J. *et al.* Large-scale high-density culture of hepatocytes in a liver microsystem with mimicked sinusoid blood flow. *J. Tissue Eng. Regen. M.* **12**, 2266-2276 (2018).
8. Linnes, M. P., Ratner, B. D. & Giachelli, C. M. A fibrinogen-based precision microporous scaffold for tissue engineering. *Biomaterials* **28**, 5298-5306 (2007).
9. Manchalwar, S. M., Anthati, V. A. & Marathe, K. V. Simulation of micellar enhanced ultrafiltration by multiple solute model. *J. Hazard. Mater.* **184**, 485-492 (2010).
10. Phan, D. T. T. *et al.* A vascularized and perfused organ-on-a-chip platform for large-scale drug screening applications. *Lab Chip* **17**, 511-520 (2017).
11. Bonfiglio, A., Leungchavaphongse, K., Repetto, R. & Siggers, J. H. Mathematical modeling of the circulation in the liver lobule. *J. Biomech. Eng.* **132**, 111011 (2010).
12. Teutsch, H. F., Schuerfeld, D. & Groezinger, E. Three-dimensional reconstruction of parenchymal units in the liver of the rat. *Hepatology* **29**, 494-505 (1999).
13. Leen, E., Cooke, T. G., Angerson, W. J. & Mcardle, C. S. Estimation of Total Hepatic Blood-Flow by Duplex Ultrasound. *Gut* **33**, 1293-1294 (1992).
14. Lautt, W. W. & Greenway, C. V. Conceptual review of the hepatic vascular bed. *Hepatology* **7**, 952-963 (1987).
15. Mareels, G., Poyck, P. P., Eloot, S., Chamuleau, R. A. & Verdonck, P. R. Three-dimensional numerical modeling and computational fluid dynamics simulations to analyze and improve oxygen availability in the AMC bioartificial liver. *Ann. Biomed. Eng.* **34**, 1729-1744 (2006).
16. Banaeiyan, A. A. *et al.* Design and fabrication of a scalable liver-lobule-on-a-chip microphysiological platform. *Biofabrication* **9**, 015014 (2017).
17. Kuntz, E. & Kuntz, H.-D. in *Hepatology Principles and Practice: History Morphology Biochemistry Diagnostics Clinic Therapy* (ed Hinrich Küster) Ch. 2, 14-29 (Springer Berlin Heidelberg, 2006).
18. Jungermann, K. & Kietzmann, T. Oxygen: modulator of metabolic zonation and disease of the liver. *Hepatology* **31**, 255-260 (2000).
19. Pacella, H. E., Eash, H. J., Frankowski, B. J. & Federspiel, W. J. Darcy permeability of hollow fiber bundles used in blood oxygenation devices. *J. Membrane Sci.* **382**, 238-242

(2011).
